# A Deeper Insight into Evolutionary Patterns and Phylogenetic History of ORF Virus through the Whole Genome Sequencing of the First Italian Strains

**DOI:** 10.3390/v14071473

**Published:** 2022-07-04

**Authors:** Elisabetta Coradduzza, Daria Sanna, Fabio Scarpa, Ilenia Azzena, Mariangela S. Fiori, Rosario Scivoli, Angela M. Rocchigiani, Roberto Bechere, Maria A. Dettori, Davide Pintus, Eloisa Evangelista, Marco Casu, Ciriaco Ligios, Giantonella Puggioni

**Affiliations:** 1 Istituto Zooprofilattico Sperimentale della Sardegna, 07100 Sassari, Italy; elisabetta.coradduzza@izs-sardegna.it (E.C.); mariangela.fiori@izs-sardegna.it (M.S.F.); rosario.scivoli@izs-sardegna.it (R.S.); angelamaria.rocchigiani@izs-sardegna.it (A.M.R.); roberto.bechere@izs-sardegna.it (R.B.); mariaantonietta.dettori@izs-sardegna.it (M.A.D.); davide.pintus@izs-sardegna.it (D.P.); ciriaco.ligios@izs-sardegna.it (C.L.); giantonella.puggioni@izs-sardegna.it (G.P.); 2 Dipartimento di Scienze Biomediche, Università di Sassari, 07100 Sassari, Italy; fscarpa@uniss.it (F.S.); iazzena@uniss.it (I.A.); 3 Dipartimento di Medicina Veterinaria, Università di Sassari, 07100 Sassari, Italy; marcasu@uniss.it; 4 Ames Polydiagnostic Group Center SRL, Casalnuovo di Napoli, 80013 Naples, Italy; elo.evangelista@gmail.com

**Keywords:** ORFV, molecular dating, Sardinia, genome types

## Abstract

Orf virus (ORFV) is distributed worldwide and is the causative agent of contagious ecthyma that mainly occurs in sheep and goats. This disease was reported for the first time at the end of 18th century in Europe but very little is currently known about the temporal and geographic origins of this virus. In the present study, the use of new Italian whole genomes allowed for better inference on the evolutionary history of ORFV. In accordance with previous studies, two genome types (S and G) were described for infection of sheep and goats, respectively. These two well-differentiated groups of genomes originated for evolutive convergence in the late 1800s in two different areas of the world (Europe for S type and Asia for G type), but it was only in the early 1900s that the effective size of ORFV increased among hosts and the virus spread across the whole European continent. The Italian strains which were sequenced in the present study were isolated on the Mediterranean island of Sardinian and showed to be exclusive to this geographic area. One of them is likely representative of the early European forms of ORFV which infected sheep and became extinct about one century ago. Such an ancient Sardinian strain may have reached the island simple by chance, where it quickly adapted to the new habitat.

## 1. Introduction

Orf virus (ORFV; family: poxviridae) is the etiological agent of the zoonotic disease contagious ecthyma (CE or ORF), also known as contagious pustular dermatitis (CPD). This virus belongs to the genus Parapoxvirus (PPV) [1] that, to date, includes four further recognized PPV species based on the classification of the International Committee on Taxonomy of Viruses (ICTV): bovine papular stomatitis virus (BPSV), grey sealpox virus, pseudocowpox virus (PCPV), and red deerpox virus (RDPV).

Contagious ecthyma was reported for the first time in sheep by Steeb in 1787 [2], and as early as the end of the 19th century, was described by Hansen (1879) [3] in goats and humans as contagious dermatitis, ORF, crusta labialis, carbuncle of the coronary band, or coupar angus [4]. It was in 1929 that Howarth [5] showed that the disease in lambs and kids in California (USA) was due to a virus which was better described for specimens from Texas (USA) by Boughton and Hardy, in 1932 [6]. The patterns of lesions connected to ORFV infection can occur in a clinical spectrum ranging from mild papular and pustolar to severe proliferative. These types of ORFV lesions are generally reported in sheep and goats, but similar kinds of lesions have also been found in other animal species [7] and humans [1,8], leading to CE being considered as a worldwide-diffused zoonotic disease [9]. In ruminants, the lesions that are provoked by ORFV infection usually involve mouth, muzzle, nostrils, gums and tongue, and occasionally feet, udders, and sporadically, gastrointestinal tract and respiratory apparatus [10]. This disease usually affects lambs and kids, thus causing their death when severe lesions avoid milk sucking from mothers. The level of morbidity from this disease is very high and, although mortality is generally rare, it can rate up to 10% in lambs [11] and to 93% in kids [12,13]. In humans, lesions are reported mainly on hands and undergo a spontaneous benign resolution in a few weeks. However, malignant cases have been also described with atypical proliferative lesions, particularly in immunocompromised individuals [7,14,15].

ORFV’s genome is a linear double stranded DNA which is 135 kb-long and encodes for 132 genes [7,16]. The central region contains highly conserved genes involved in viral replication and in the assembly of the viral structure, while terminal regions are more variable and include genes for virulence and immunomodulation [7,17]. The occurrence of high levels of nucleotide variation for the terminal regions of ORFV may be connected to the phenomenon of reinfection typical of this virus that also appears as a product of the regulation acted by viral genes on the host innate immune response [14]. In such a context, many studies focused their attention on the immunomodulatory action of specific ORFV genes with the aim to shed light on the complex processes involved in viral pathogenesis [18,19].

Only two studies, which were performed to describe novel ORFV genomes isolated in China [20] and France [21], provided phylogenetic inferences based on ORFV whole genomes. The first study [20] was performed on nine genomes and evidenced the occurrence of a well-supported genetic structuring between the strains isolated from sheep and those from goats. Consistently, results obtained in the second study [21], where twelve genomes from almost all the continents were used, evidenced a genetic differentiation depending on whether the virus host was a goat or a sheep. This latter study also described, for the first time, an ORFV genome isolated from a human after infection from sheep. A genetic affinity between genomes from human infections was also found in this study. These authors suggested the importance to analyze a larger number of ORFV whole genomes to confirm the possible occurrence of two types of ORFV (from sheep vs. from goats) and shed new light of the level of genetic variation associated with the host species.

In such a context, in the present study, seven new ORFV whole genomes from the Mediterranean island of Sardinia (Italy) were isolated from sheep and goats and merged with all of the ORFV genomes currently available on public databases to perform a high-resolution phylogenetic analysis of the virus.

A similar phylogenetic deep inference was previously performed for samples from all over the world based on the ORFV gene, encoding the dsRNA-binding protein (*VIR*) [22]. Results evidenced a high worldwide mutational viral evolutionary rate, along with a well-supported genetic divergence between the viral strains isolated from sheep and those from goats.

The aim of this study is to provide new insight into the evolutionary history of ORFV based on the analysis of whole genomes, to better understand the temporal origin of its strains with corresponding patterns of distribution, and to make inferences on the possible occurrence of two different genome types of ORFV associated with sheep and goat infections.

## 2. Materials and Methods

### 2.1. Sampling

Samples were collected, between May 2017 and March 2021, in five different Lacaune and Sarda breed sheep flocks and in one Sarda goat herd (see Table 1 for details). ORFV infection evidenced by clinical signs in individuals was confirmed by virological and molecular analyses of biological samples. Clinical CE-outbreaks have been detected and reported by private practitioners and veterinary public health components involved in the present research, during their diagnostic activity. Sampling collection protocols were as reported by Coradduzza et al., 2021 [22]. Viral DNA was extracted from lesions isolated in five infected sheep and two infected goats (see Table 1 for details on the samples).

### 2.2. Virus Isolation

ORFV was isolated on Vero cells from homogenates tissues of ovine and caprine mild and severe-type lesions according to the methodologies reported by Coradduzza et al., 2021 [22]. In particular, a total of 0.5 g of each tissue sample was homogenized in 5 mL (10% *w*/*v*) of DMEM medium with the use of the following antibiotics: 400 UI/mL penicillin, 400 μg/mL streptomycin, 300 μg/mL gentamicin, and 2.5 μg/mL amphotericin B. The suspension obtained was centrifuged at 1000× *g* for 15 min and used for the infection of Vero cells using cells from the 100th to 120th passage (BSCL86 ATCC, Istituto Zooprofilattico Sperimentale della Lombardia e dell’Emilia Romagna, Italy). Twelve-well plates of cells in DMEM were prepared with the addition of 10% (*v*/*v*) FBS and then incubated for 1 h in a thermostat at 37 °C in an atmosphere of 5% CO_2_. After 18–24 h (80–90% confluence), the medium was removed and the cells were incubated with 0.5 mL of tissue homogenate under the same incubation conditions. Furthermore, 3 wells without the virus, used as a negative control, along with 3 wells containing the virus, were also prepared; after incubation, the cells were washed three times with 1× PBS and the new DMEM with antibiotic, and fetal bovine serum was added (final concentration 100 UI/mL penicillin, 100 µg/mL streptomycin, and 0.5 µg/mL amphotericin B with 2% (*v*/*v*) FBS). The cytopathic effect (CPE) was checked daily, when it was detected, or on the 5th day of culture, the material was freeze-thawed three times, collected, and centrifuged at 200× *g* for 10 min. The collected supernatant was stored at 80° for future use.

### 2.3. Viral DNA Extraction, Sequencing and Genome Assembly

After centrifugation at 10,000× *g* for 1 min at 4 °C, viral DNA was extracted from cell culture supernatant using a QIAmp UltraSens Virus Kit (Qiagen, Hilden, Germany), as described in Fiori et al. [23] to perform the whole genome sequencing using Illumina platform.

DNA quantification was performed using an Epoch microplate spectrophotometer (BioTek, Winooski, VT, USA) and a Qubit 2.0 Fluorometer (Thermo Fisher Scientific, Waltham, MA, USA), in agreement with the manufacturer’s instructions. Viral DNA libraries were prepared using Nextera DNA Flex Library Prep kit (Illumina Inc., San Diego, CA, USA). The samples were sequenced with Novaseq 6000 (Illumina) generating paired-end reads 2 × 150 (AMES Group, Centro Polidiagnostico Strumentale S.r.l, Napoli, Italy). The resulting FASTQ files, containing 5,000,000 reads, were tested for quality using the tool fastqc implemented in the command line package FASTX-Toolkit v0.7 [24], which was also used for removing sequencing adapters (command line: fastx_clipper -e 0.1 -a CTGTCTCTTATACACATCT -l 15) and trimming too short and low quality sequences (command line: fastq_quality_filter -v -m 30 -Q 33 -q 25 -p 50). Assembly was performed using Bowtie 2 v2.4.2 (Johns Hopkins University, Baltimora, MD, USA) [25,26], and a new filter was applied for reads whose QSEQ filter field was non-zero. Reads were mapped against the reference genome NC_005336.1 (this genome is the only one branded as reference genome for ORFV in NCBI Virus portal) using an end-to-end read alignment (basic command line: bowtie2 [options]* -x <bt2-idx> {−1 <m1> −2 <m2> | -U <r> | --interleaved <i> | --sra-acc <acc> | b <bam>} -S [<sam>]). A de novo assembly was also performed to use an approach as complete as possible, using the software SPAdes v3.15.3 (Center for Algorithmic Biotechnology, St Petersburg, Russia) [27] (command line: --outdir out --cpus 6 --ram 30 --R1 fastq_r1.fastqsanger --R2 fastq_r2.fastqsanger --trim --namefmt contig%05d --depth 100 --nocorr --minlen 0 --mincov 2 --assembler spades) by following Scarpa et al. [28] and Piredda et al. [29]. Scaffolds were subsequently oriented with the reference genome NC_005336.1 by using Unipro UGENE v.35 (Unipro Center for Information Technologies, Novosibirsk, Russia) [30].

### 2.4. Phylogeny, Molecular Dating and Evolutionary Rate

Seven whole genome sequences were obtained for ORFV in the present study. The whole dataset (*n* = 26) was aligned using the Clustal Omega package [31] (available at https://www.ebi.ac.uk/Tools/msa/clustalo/; accessed on 15 April 2022) after editing by means of Unipro UGENE v.35 [29], and subsequently deposited in GenBank (see Table 2 for accession numbers).

These Sardinian sequences were included in a large dataset containing all the ORFV whole genomes available on GenBank to date (see Table 2 for details and references). This dataset included a total of 26 sequences; 7 from the present study (Italy), 2 from Germany, 1 from France, 10 from China, 1 from India, 1 from New Zealand, and 4 from United States of America (USA) (see Table 2 for details).

To identify potential genetic clusters within the dataset and to determine the dissimilarity represented by the genetic variability among genomes, a PCoA (principal co-ordinate analysis) was performed using GenAlEX 6.5 [38]. PCoA reconstruction was based on a genetic pairwise p-distance matrix.

The jModeltest 2.1.1 software [39] was used to find the best probabilistic model of genome evolution with a maximum likelihood optimized search. MrBayes 3.2.7 [40] was used to carry out a Bayesian phylogenetic analysis, setting nst = 6, rates = invgamma, and ngammacat = 4 as model parameters. Two independent runs, with 4 Metropolis-coupled Markov chain Monte Carlo (MCMC) chains (1 cold and 3 heated chains), were run synchronously for 5 million generations, sampling trees every 1000 generations. The first 25% of sampled trees were discarded for burn-in. The average standard deviation of split frequencies (which should approach 0) was verified checking the convergence of chains [40] and the potential scale reduction factor (which should be approximately 1) [41] following Scarpa et al. [42].

Phylogenetic trees were visualized and edited using the FigTree 1.4.1 software (available at http://tree.bio.ed.ac.uk/software/figtree/; accessed on 15 May 2022).

When available, the collection date of the samples (at least month and year were necessary) were used to set molecular dating which was performed by means of a Bayesian approach under the MCMC algorithm as implemented in Beast 1.10.4 software [43]. Only the genomes whose sample collection date was known were included in this analysis. Both strict and uncorrelated log-normal relaxed clock models were tested with fast runs of 100 million generations to identify the best clock model to perform the dating analyses. Selection was made comparing Bayes factor values using Tracer 1.7 software [44]. All available demographic models (both parametric and nonparametric) were also tested. The phylogenetic time-scaled (ultrametric) trees and the evolutionary rates were co-estimated—after the selection of the Bayesian skyline demographic model under the uncorrelated log-normal relaxed clock model—running 500 million generations, with samplings repeated every 50,000 generations. The resulting log files were inspected using Tracer 1.7 software [44]. Only values of ESS (effective sample size) ≥200 were accepted. The maximum clade credibility tree was drawn, visualized, and annotated by means of the software TreeAnnotator (Beast package) and FigTree 1.4.1, respectively. Beast software was also used to perform further runs under the coalescent Bayesian skyline demographic model [45] to estimate the evolutionary rates for ORFV strains isolated in sheep and goats. All phylogenetic/phylodinamic runs were carried out using the CIPRES Science Gateway (Cyberinfrastructure for Phylogenetic Research available at https://www.phylo.org/index.php/; accessed on 8 May 2022) [46] with a total effort of about 5000 CPU hours of computation.

## 3. Results

Assembly performed for the Italian ORFV strains with the reference genome (NC_005336) provided seven new whole genomes. See Table A1 and Table A2, provided in Appendix A, for details regarding each new genome.

### Phylogeny, Molecular Dating, and Evolutionary Rate

In the present study, a dataset including 26 ORFV whole genomes that were 137,151 bp long, was analyzed. The sequences were isolated in all continents with the only exception being Africa (see Table 2 for details). The nucleotide frequency analysis carried out on the whole dataset reveals that conserved sites amount to 118,671 (86.5%), variable sites amount to 18,478 (13.5%), parsimony informative sites amount to 11,801 (8.6%), and singletons amount to 6677 (4.9%).

**Figure 1:**
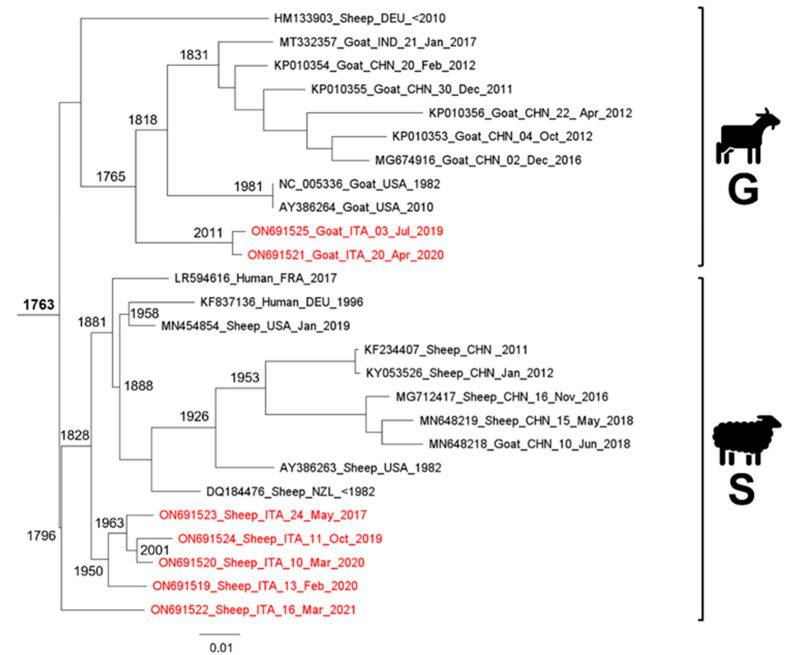
Bayesian phylogenetic tree based on all ORF virus whole genomes used in the present study. The Italian whole genomes from Sardinia which were obtained in the present study are indicated with a red font. All the nodes of the tree are fully supported and values of posterior probabilities for all the nodes are = 1. Capital letters on the right indicate the genetic clusters described in the text. The year indicated at the main node represent the coalescent time of the cluster and was inferred according to the molecular dating estimates (years before 16 March 2021) reported in the ultrametric tree (Figure A1).

**Figure A1:**
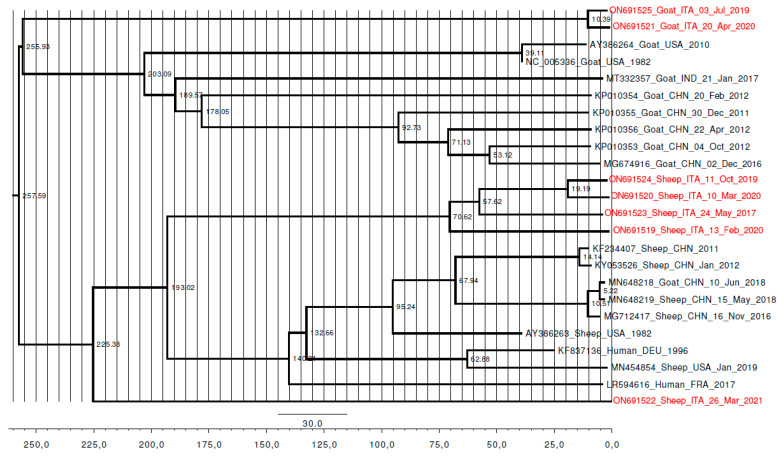
Bayesian phylogenetic time-scaled maximum clade credibility tree based on all ORF virus whole genomes used in the present study. All nodes of the tree are highly supported by values of posterior probabilities ≥0.95. The Sardinian sample codes are reported in red font. The codes of sequences report the GenBank accession number, the country acronym, and the presumptive year of isolation. The numbers at the nodes refer to the confidence interval (C.I.) 95% highest posterior density (HPD) for the dating of the cluster. Capital letters on the right refer to the genetic clusters indicated in Figure 1. Horizontal bar indicates the years before 26 March 2021 on a temporal scale.

The phylogenetic mid-point tree analysis evidenced the occurrence of two fully-supported genetic clusters (cluster S and cluster G in Figure 1). The Bayesian phylogenetic time-scaled maximum clade credibility tree (ultrametric tree), used for the molecular dating, is provided in Appendix A (as Figure A1) with the confidence interval (C.I.) at 95% of the highest posterior density (HPD) indicated for each coalescence time estimate. Strains whose phylogenetic position was puzzling in the phylogenetic tree (GenBank # HM133903, MN648218) were excluded from this latter analysis. 

Furthermore, only genomes whose sample collection date was available were used to perform the molecular dating (two genomes were removed from the dataset used for molecular dating: GenBank # HM133903, DQ184476). 

Results obtained from the ultrametric tree (Figure A1) are consistent with those obtained from the phylogenetic tree (Figure 1), with a few discrepancies due to the lack of three genomes in the dataset analyzed, as reported above. The coalescence time at each node of the phylogenetic tree was inferred according to the molecular dating estimates obtained with the ultrametric tree.

The common ancestor of all the ORFV strains present in the analyzed dataset, which corresponds to the root of the tree, dates back to 257.59 years before 2021 (i.e., the end of the year 1763). The cluster G of the tree includes only ORFV genomes isolated from goats with a single exception. Indeed, within this group of sequences, a genome isolated in a sheep from Germany (GenBank # HM133903) is also present and sets in a basal position, as an ancestral and quite divergent strain, outside the ingroup of genomes from Italy, India, China, and USA. For this strain, the date of collection is unknown (probably before year 2010), and for this reason it was not possible to estimate its divergence time (indeed, this genome was not included within the dataset used to construct the ultrametric tree, see Figure A1). Within the ingroup, which dates back to 255.93 years before 2021 (i.e., the middle of the year 1765), the genomes from Sardinia (Italy) (two kids) that were isolated in 2019 and 2020, set as an external sub-cluster that originates 10.39 years before 2021 (i.e., the final part of the year 2010). The other genomes of cluster G were grouped in a sub-cluster that dates back to 203.09 years before 2021 (i.e., the beginning of the year 1818), which includes two sister clades. The first sister clade dates back to 189.57 years before 2021 (i.e., the end of the year 1831) and includes only samples from Asia (China and India) isolated between the years 2011 and 2017. The second sister clade, which originates 39.11 years before 2021 (i.e., the middle of the year 1981), includes two genomes from USA isolated in 1982 and 2010.

The cluster S of the phylogenetic tree includes only sequences isolated from humans (infected from sheep) and sheep, with the only exception of a genome which was isolated in a goat from China in 2018 (GenBank # MN648218). Within cluster S, which dates back to 225.38 years before 2021 (i.e., the beginning of the year 1796), a sequence isolated in a lamb from southern Sardinia (Italy) in 2021 sets in a basal position, external to the ingroup that contains all the other sequences of the cluster. Inside the ingroup that originates 193.01 years before 2021 (i.e., the beginning of the year 1828), two sister clades are present. The first sister clade dates back to 140.21 years before 2021 (i.e., the beginning of the year 1881) and includes genomes isolated from humans in Europe (France and Germany) and from sheep in Asia (China), USA, and Oceania (New Zealand). The two genomes isolated from humans in the years 1996 and 2017, along with a genome isolated from a sheep in USA in 2019, set in an external position within the sister clade. The remaining genomes from China, USA, and New Zealand were isolated between 1982 (maybe earlier) and 2018. In particular, within this last group of genomes, the two external strains from New Zealand and USA, which were isolated in 1982 (USA) or maybe earlier (New Zealand), were the unique strains that were not collected in 2000s, and the sub-cluster of genomes from Asia (China) dates back to 67.94 years before 2021 (i.e., the middle of the year 1953).

The second sister clade of cluster S dates back to 70.62 years before 2021 (i.e., the end of the year 1950) and includes only sequences from Sardinia (Italy) collected between 2017 and 2020. In particular, within this Sardinian clade, a sequence isolated in an adult from the northern part of the island in 2020 sets in a basal position, while all the other strains (also isolated in the northern of the island), collected from a lamb and two adults, between the 2017 and 2020, grouped together in a sub-cluster that originates 57.62 years before 2021 (i.e., the end of the year 1963). Within this sub-cluster, the two sequences collected in 2019 and 2020 belong to a group that dates back to 19.19 years before 2021 (i.e., the end of the year 2001).

**Figure 2:**
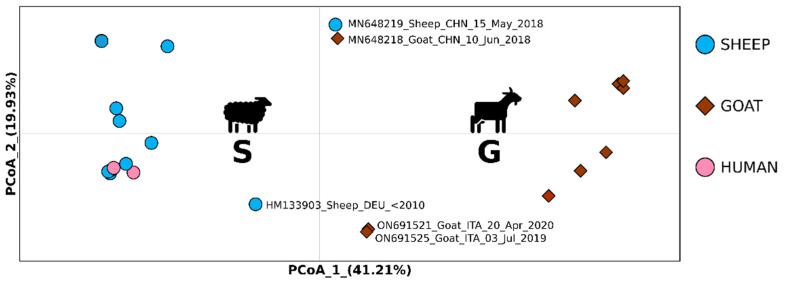
Principal coordinates analysis performed on all ORF virus whole genomes dataset. Bidimensional plot shows the genetic differentiation among specimens due to the nucleotide substitutions per site found in the dataset. Capital letters in the plot indicate the genetic clusters described in the text.

The PCoA (Figure 2) performed to evidence the occurrence of genetic clusters among the genomes analyzed, was able to explain 61.14% of the variability (PCoA1/X axis: 41.21%, PCoA2/Y axis: 19.93%). Results were consistent with those obtained from the phylogenetic tree analysis, thus evidencing the occurrence of divergence between ORFV strains isolated from sheep and humans and ORFV strains isolated from goats (cluster S and cluster G, respectively, in Figure 2). Furthermore, it is interesting to note that in the PCoA graphic, the genome isolated in a sheep from Germany, which sets in a basal position of cluster G (goats) in the phylogenetic tree, is included as an outlier within the variability of strains isolated from sheep. In accordance with the phylogenetic tree analysis, the genomes from Sardinia (Italy) isolated in goats, set as outliers within the variability of strains isolated from goats. The two genomes from China isolated from a sheep and a goat, which are included as a highly divergent group within cluster S of phylogenetic tree, could be considered as divergent strains outside the variability of the two groups of genomes evidenced by PCoA.

Under the Bayesian skyline lognormal uncorrelated relaxed clock model, the viral evolutionary mean rate calculated for the ORFV whole genome dataset was estimated to be 1.324 × 10^−6^ substitution/site/year, with a C.I. 95% HPD of 7.488 × 10^−10^–5.439 × 10^−6^ (1.324 × 10^−6^ [7.488 × 10^−10^–5.4396 × 10^−6^]). The viral evolutionary mean rate was also calculated for clusters S and G of phylogenetic tree and principal co-ordinates analyses; the strains whose sampling collection dates were unknown, along with those whose phylogenetic position was puzzling (GenBank # HM133903, MN648218), were excluded from the datasets. The two values were estimated to be 4.901 × 10^−4^ substitution/site/year, with a C.I. 95% HPD of 1.294 × 10^−5^–1.026 × 10^−3^ (4.901 × 10^−4^ [1.294 × 10^−5^–1.026 × 10^−3^]) and 3.672 × 10^−6^ substitution/site/year, with a C.I. 95% HPD of 1.013 × 10^−8^–1.253 × 10^−5^ (3.672 × 10^−6^ [1.013 × 10^−8^–1.253 × 10^−5^]) for cluster S and cluster G, respectively.

## 4. Discussion

The present study allowed for a 37% increase in the number of whole genomes available for ORF virus from all over the world. In particular, we provided the first Italian sequences for this virus, thus expanding the number of European countries for which ORFV genomes are known. According to Chi et al. [20], who were the first to report a great genetic divergence between goat ORFVs and sheep ORFVs, and Andreani et al. [21], who suggested that more indepth research was needed to understand worldwide ORFV distribution, the present research expands the knowledge on the evolutionary history of ORFV, thus providing the first hints of the temporal and geographic origins of the virus and evidencing the occurrence of two highly-differentiated types of genomes. They are the lineages of ORFV associated with the infection of goats (type G genomes) and sheep (type S genomes). The strong genetic divergence found between these two genome types could be related to the general lack of transmission of the ORFV infection between sheep and goats that would have prevented the occurrence of viral recombination and genetic similarity, among types S and G strains. Indeed, although this virus can be transmitted among hosts by direct contact with tissue lesions or fomites, thus infecting animals through skin cuts and abrasions, in general it is reported to be transmitted from wild to domestic goats, but not to domestic sheep [47]. Furthermore, in accordance with Chi et al. [20], who found that ORFV genomes isolated from goats are more similar to each other than those isolated from sheep, the evolutionary rate calculated for type G genomes in the present study was lower than that calculated for type S genomes, suggesting the occurrence of a stronger selective pressure acting on the viral strains that infect sheep along with a higher capability of type S genomes to quickly adapt to environmental changes. A possible explanation for this finding may involve the immune response of sheep that could be highly reactive towards ORFV as a consequence of extensive vaccination campaigns, thus prompting the evolutive rate of the virus with the occurrence of frequent viral recombination among type S genomes.

Furthermore, the evolutionary mean rate calculated for the ORFV type G genomes was similar to the value obtained in the present study when the strains from all hosts (goats, sheep and humans) were considered together. However, the evolutionary mean rate calculated for the ORFV type S genomes was higher and more similar to the value obtained by Coradduzza et al. [22] for the ORFV *VIR* gene which was isolated in hosts from all continents. This latter finding suggests a possible connection (that must be supported by further specific genetic studies) between the occurrence of contagious ecthyma in sheep and the expression of the gene encoding the dsRNA-binding protein in the viral strains that infect these animals.

In accordance with Andreani et al. [21], in the present study, different ORFV strains isolated after a human infection from sheep were closely related to one another and belonged to an external heterogeneous genetic sub-group within the type S genomes clade. Interestingly, considering that human-to-human transmission has never been reported [48], with only few exceptions which involve direct contacts with other human lesions or fomites [49], and according to Andreani et al. [20], who suggested the use of many genomes to evidence the occurrence of ORFV variants related to the strains isolated from humans that may be able to again cross species barriers; in the present study an ORFV strain (GenBank # MN454854) from a cell culture infected with contagious ecthyma vaccine in Texas (USA) in 2019 might represent a viral strain that can easily produce new spillover between sheep and humans. These events are frequent during the whole evolutive history of zoonoses and represent the main reason for which infections originating in wildlife require constant monitoring (see Scarpa et al., 2021 and references therein) [50]. In such a context, although contagious ecthyma is considered as an occupational disease in “human risk populations”, such as shepherds, sheep shearers, butchers, and veterinary surgeons, studies on a large number of ORFV genomes isolated from animals and humans are needed to better infer the evolutionary patterns occurring among types S lineages and to better describe the characteristics of the viral strains that infect sheep and can be easily transmitted to humans in specific conditions (i.e., direct or indirect contact with infected animals). In fact, considering that the ORFV strains isolated in farmers infected by sheep in the present study resulted in genetically divergence from those that are common among sheep, there is a strong possibility that only specific viral strains, with high levels of virulence, are able to infect humans even when environmental conditions make the possibility of ORFV transmission extremely low.

In the phylogenetic tree (Figure 1) of the present study, an ORFV strain isolated from a sheep in Germany (GenBank # HM133903) before 2010, which was considered as a puzzling outgroup genome by Andreani et al. [21], was included within the G (goats) cluster, and thus confirmed the peculiar nature of this viral allelic variant (as evidenced in the previous studies) that could be representative of an ancestral form of ORFV. Notably, this same viral strain in the PCoA (Figure 2) of our research clustered within the type S genomes isolated from sheep evidencing that this latter analytical approach could provide a more representative description of the genetic structuring among viral genomes. Indeed, PCoA, which was reconstructed on a p-distances matrix (without information on evolutionary models) is able to depict divergences or similarities based on differences in the genomic nucleotide composition of strains.

The common ancestor to all viral strains included in the present study (see also Figure 1 for molecular dating of the tree clusters), which could be considered as a proto-ORFV form not yet well-differentiated in the present types S and G ORFV, dates back to the end of 1763. This value represents a molecular dating which is consistent with the first description of contagious ecthyma in European sheep some years later by Steeb in 1787 [2], and in European goats and humans in 1879 [4]. In the present study, types S and G ORFV genome groups are almost coevals (1796 and 1765, respectively), and the rise of these two well-differentiated and contemporary genetic clades may represent a typical case of multiregional geographic origin by evolutive convergence of viral strains in different host species (indeed, identical pathological phenotypes/patterns of clinical lesions are reported for sheep and goats) (see e.g., Sackman et al., 2017 and references therein) [51]. In particular, regarding the type G strains, they may represent the first group of lineages that differentiated, thus suggesting that the early form of ORFV associated with the development of contagious ecthyma originated from viruses infecting goats. Their geographic origin could be tentatively placed in Asia, which currently represents the continent where the highest and the oldest (year 1831) level of genetic variability was evidenced in our analyses.

Furthermore, considering that contagious ecthyma was described for the first time in goats from European farms in 1879 [4], ORFV’s type G strains may have taken about one century (from 1765 to 1879) to spread from Asia toward Europe, thus increasing its population size among hosts. This virus may have reached Europe, not only transported by live hosts involved in animal trade, but also with goods of animal origin along the commercial routes between the two continents. Indeed, ORFV remains viable on the wool of animals (infected and recovered) and contaminated materials for long periods, enabling indirect transmission to new susceptible hosts [52,53]. In general, this virus remains viable on the host’s wool and survives for about one month after the lesions have healed, but it can also be carried by clinically normal sheep. Furthermore, ORFV was reported to survive in laboratory lesion tissue samples for up to 12 years [48], and for up to 17 years in natural environments with a dry climate [14] as the trade commercial routes between Asia and Europe likely were in the 1800s.

Within the type G clade, two sub-lineages are exclusive to the USA and Italy (Sardinia). In particular, the Italian allelic variants isolated in Sardinia may be considered as representative of a group of lineages exclusive to the island that originated in loco in 2011. Indeed, contagious ecthyma was first reported on this island in the early 1990s, according to a booklet edited by the Zooprophylactic Institute of Sardinia (Italy) as part of a health education project, and these recently-differentiated strains might have diverged from the first founders under the selective pressure of the new island habitat. The molecular dating (2011) provided for the Sardinian type G ORFV genomes cluster is consistent with the value (years 2009 and 2012) obtained by Coradduzza et al. [22] for strains isolated from Sardinian goats, on the basis of the ORFV *VIR* gene.

Our results suggest that the ORFV type S genomes clade is 31 years younger than type G and differentiated in the year 1796. This finding is consistent with the first report of contagious ecthyma in European (maybe Danish) sheep by Steeb in 1787 [2], suggesting that the early form of ORFV, associated with the development of contagious ecthyma in sheep, originated in northern Europe. However, with the only exception being a Sardinian, likely ancestral strain (GenBank # ON691522), all the type S genomes included in the present study share a common ancestor which dates back to 1828. This latter molecular dating may correspond to the beginning of the ORFV expansion across Europe and Asia and it matches with the first detailed description of contagious ecthyma in European sheep provided as early as 1890 by Wallay [9]. In this case, considering the young age of the Asian (Chinese) cluster (year 1953) of type S genomes, ORFV may have taken about a century (or even less) to spread from Europe toward Asia.

This evolutionary scenario in consistent with the extinction of most of the early European ORFV strains, as only those strains which performed better adaptative variation to the habitat are the ancestors of the genomes which expanded at the end of 19th century and, later in the 1920s, spread across the whole continent as suggested by several reports of contagious ecthyma in many countries [52,53,54,55].

The Sardinian ancestral type S strain (GenBank # ON691522) was isolated in 2021 from a lamb belonging to the Sarda sheep breed that lived on a farm in the southern part of the island. In particular, in accordance with personal communications obtained by these authors from the farm’s owners, new heads were never introduced into their livestock and parent animals always either lived on the same farm or came from neighboring areas. In such a context, the Sardinian Sarda sheep breed was selected on the island in 1927/28 (in particular, in 1928 the first aries and the first sheep were registered in the Herd Book, they were owned by the itinerant Chair of Agriculture of Cagliari directed by Professor Francesco Passino) from heads arriving from the European mainland. Therefore, the ancestral ORFV Sardinian strain isolated in the present study could be a direct descendant of the early ancient ORFV strains that probably originated in northern Europe at the end of 18th century and then became extinct. This likely uncommon form of ORFV may be present in Sardinia simply by chance according to a typical effect of genetic drift and may have survived on the island as, after its arrival, it became well-adapted to the new environment. In such a context, this ORFV strain might be considered as a relic of the first ORFV variants that originated early on European mainland and is worthy of properly investigation to better understand the origins of this virus.

Conversely, the other Sardinian ORFV strains included within the type S genomes clade of the present study were isolated in the northern part of the island and they belong to a divergent genetic group that dates back to 1950 and includes viral allelic variants that originated in 1963 and 2001. The strains that have differentiated in the 21st century may be exclusive to the island, specifically the northern area. Another point of interest is that the molecular dating provided by the present research for the common ancestor of the ORFV genome strains isolated from sheep in Sardinia (year 1950) is quite consistent with the estimation reported by Coradduzza et al. [22] based on the ORFV *VIR* gene (year 1925). The small discrepancy could be related to the different molecular datasets used to perform molecular dating (whole genome 137,151 bp vs. *VIR* gene 382 bp). In general, the results obtained in the present study for ORFV strains isolated from heads living in northern and southern Sardinia, suggest that different strains of this virus arrived on this Mediterranean island during distinct periods of the 20th century. These highly divergent allelic variants were introduced in different areas of the island where various kinds of selective pressure might have originated new private and endemic ORFV variants. As a consequence, and as previously reported by Coradduzza et al. [22], the enclosed nature of Sardinia may have prompted the growth of a strong genetic differentiation among viral strains from different geographic areas. In the future, a higher number of ORFV whole genomes isolated from Sardinian hosts could help to shed further light of the evolutionary history of ORFV on the island and to provide evolutive predictive models of the distribution of this virus for areas of the world with similar environmental and selective conditions.

## 5. Conclusions

In conclusion, further analyses on many ORFV whole genomes are needed to corroborate the multiregional geographic origin in the late 18th century for ORFV strains infecting sheep (type S) and goats (type G), in Europe and Asia, respectively, as it is suggested in the present study.

## Figures and Tables

**Table 1:** Sampling plan. The table reports metadata on the Italian ORF virus strains resulting from epidemics on Sardinian farms analyzed in the present study. GenBank accession number: GB #.

Sample ID	GB #	Country	Collection Site	Co-Ordinates	Collection Date	Host
S21	ON691523	Italy	Sardinia-Sassari	LAT: 40.77916 LON: 8.41332	24 May 2017	Sheep
S30	ON691525	Italy	Sardinia-Sassari	LAT: 40.60107 LON: 8.34540	3 July 2019	Goat
S27	ON691524	Italy	Sardinia-Sassari	LAT: 40.76113 LON: 9.0016	11 October 2019	Sheep
S6	ON691519	Italy	Sardinia-Sassari	LAT: 40.60107 LON: 9.0016	13 February 2020	Sheep
S10	ON691520	Italy	Sardinia-Sassari	LAT: 40.58005 LON: 9.09239	10 March 2020	Sheep
S15	ON691521	Italy	Sardinia-Sassari	LAT: 40.64278 LON: 8.89063	20 April 2020	Goat
S19	ON691522	Italy	Sardinia-Cagliari	LAT: 39.28786 LON: 9.25448	26 March 2021	Sheep

**Table 2:** Metadata of ORF virus whole genomes already deposited in GenBank and used in the present study.

Sample ID	GB #	Country	Collection Date	Host	Reference
NA1/11	KF234407	China	26 October 2011	Sheep	Li et al., 2013 [10]
OVHN3/12	KY053526	China	January 2012	Sheep	Not available
SY17	MG712417	China	16 November 2016	Sheep	Not available
CL18	MN648219	China	15 May 2018	Sheep	Not available
NP	KP010355	China	30 December 2011	Goat	Chi et al., 2015 [20]
GO	KP010354	China	20 February 2012	Goat	Chi et al., 2015 [20]
SJ1	KP010356	China	22 April 2012	Goat	Chi et al., 2015 [20]
YX	KP010353	China	04 October 2012	Goat	Chi et al., 2015 [20]
NA17	MG674916	China	02 December 2016	Goat	Not available
GZ18	MN648218	China	10 June 2018	Goat	Not available
MP	MT332357	India	21 January 2017	Goat	Nayak et al., 2020 [32]
NZ2	DQ184476	New Zealand	Unknown ≤ 1982	Sheep	Mercer et al., 2006 [33]
TVL	MN454854	USA	January 2019	Sheep	Heare et al., 2019 [34]
OV-IA82	AY386263	USA	1982	Sheep	Delhon et al., 2004 [35]
OV-SA00	NC005336	USA	1982	Goat	Delhon et al., 2004 [35]
OV-SA00	AY386264	USA	2010	Goat	Delhon et al., 2004 [35]
IHUMI-1	LR594616	France	2017	Human	Andreani et al., 2019 [21]
D1701	HM133903	Germany	Unknown ≤ 2010	Sheep	McGuire et al., 2010 [36]
B029	KF837136	Germany	1996	Human	Friederichs et al., 2013 [37]

## Data Availability

The sequences of the ORFV whole genomes obtained during the present study are openly available in the GenBank nucleotide sequence database. Accession numbers: ON691519–ON691525. See Table A2 in Appendix A for more information on the data accessibility.

## Appendix A

**Table A1:** Information regarding the seven ORF virus whole genome sequences isolated in hosts from the Mediterranean Italian island of Sardinia during the present study. The length of genome does not include gaps.

		S6	S10	S15	S19	S21	S27	S30
**Culture isolate**		Sheep crusted lesion	Lamb crusted lesion	Kid proliferative lesion	Lamb crusted lesion	Sheep crusted lesion	Sheep crusted lesion	Kid crusted lesion
**Genome length**		128,110 nt	128,257 nt	129,776 nt	131,660 nt	129,506 nt	129,858 nt	130,411 nt
**n° High quality reads mapped**		1,243,066	7959	4681	277,763	6570	25,512	300,184
**% Mapped genome**		91.5	91.6	92.7	94.1	92.5	92.8	93.2
**Average per base coverage**		1453.3×	9.3×	16.9×	315×	7.6×	31.6×	343.3×
**Base composition**		A: 17.6%	A: 17.8%	A: 17.8%	A: 17.8%	A: 17.8%	A: 17.8%	A: 17.9%
		C: 32.5%	C: 32.3%	C: 32.2%	C: 32.2%	C: 32.3%	C: 32.3%	C: 32.3%
		G: 32.6%	G: 32.2%	G: 32.2%	G: 32.2%	G: 32.2%	G: 32.3%	G: 32.3%
		T: 17.3%	T: 17.7%	T: 17.7%	T: 7.7%	T: 17.6%	T: 17.6%	T: 17.5%
**Contigs information ***	Max length	69,576 nt	5175 nt	8125 nt	69,407 nt	8126 nt	9279 nt	488,393 nt
	Min length	104 nt	112 nt	92 nt	92 nt	92 nt	92 nt	92 nt
	Total number	184 contigs	2245 contigs	778 contigs	1041 contigs	746 contigs	4474 contigs	1022 contigs
**% Identity with the reference genome NC_005336 ****		97%	98%	98%	97%	97%	97%	98%

* The contigs information row refers to de novo assembly. ** The percentage of identity has been inferred by using the blast tool implemented in the NCBI Virus portal.

**Table A2:** Information on the data accessibility on the NCBI database.

Sample ID	GenBank	Biosample	Sequence Read Archive (SRA)	BioProject
S21	ON691523	SAMN28405497	SRR19732912	PRJNA838097
S30	ON691525	SAMN28405499	SRR19732910	
S27	ON691524	SAMN28405498	SRR19732911	
S6	ON691519	SAMN28405493	SRR19732916	
S10	ON691520	SAMN28405494	SRR19732915	
S15	ON691521	SAMN28405495	SRR19732914	
S19	ON691522	SAMN28405496	SRR19732913	
